# Design and Performance of a Pinned Photodiode CMOS Image Sensor Using Reverse Substrate Bias †

**DOI:** 10.3390/s18010118

**Published:** 2018-01-03

**Authors:** Konstantin D. Stefanov, Andrew S. Clarke, James Ivory, Andrew D. Holland

**Affiliations:** Centre for Electronic Imaging, The Open University, Walton Hall, Milton Keynes MK7 6AA, UK; andrew.c@engineer.com (A.S.C.); James.Ivory@open.ac.uk (J.I.); Andrew.Holland@open.ac.uk (A.D.H.)

**Keywords:** CMOS image sensors (CIS), pinned photodiode, full depletion, reverse bias, thermionic emission

## Abstract

A new pinned photodiode (PPD) CMOS image sensor with reverse biased p-type substrate has been developed and characterized. The sensor uses traditional PPDs with one additional deep implantation step to suppress the parasitic reverse currents, and can be fully depleted. The first prototypes have been manufactured on an 18 µm thick, 1000 Ω·cm epitaxial silicon wafers using 180 nm PPD image sensor process. Both front-side illuminated (FSI) and back-side illuminated (BSI) devices were manufactured in collaboration with Teledyne e2v. The characterization results from a number of arrays of 10 µm and 5.4 µm PPD pixels, with different shape, the size and the depth of the new implant are in good agreement with device simulations. The new pixels could be reverse-biased without parasitic leakage currents well beyond full depletion, and demonstrate nearly identical optical response to the reference non-modified pixels. The observed excessive charge sharing in some pixel variants is shown to not be a limiting factor in operation. This development promises to realize monolithic PPD CIS with large depleted thickness and correspondingly high quantum efficiency at near-infrared and soft X-ray wavelengths.

## 1. Introduction

Pinned photodiode (PPD) monolithic CMOS image sensors (CIS) are the dominant devices in today’s high performance and consumer imaging due to several key characteristics. The use of charge transfer between the PPD and the sense node separates the functions of charge collection and charge-to-voltage conversion [1] and allows the sense node to be optimized separately and to be much smaller than the PPD. Optimal correlated double sampling can be implemented for suppression of kTC noise, and together with the very low sense node capacitance makes it possible to routinely achieve sub-electron read noise [2]. In addition, the dark current in PPD pixels is very low because of the nearly complete suppression of the surface electron-hole generation by the shallow pinning layer [3], which also helps to significantly reduce the image lag [4].

For applications requiring high quantum efficiency (QE) in the near-infrared (NIR) and soft X-ray (<10 keV) bands, the active sensor volume should be tens or even hundreds of micrometers thick due to the large absorption length of silicon [5]. To prevent deterioration of the modulation transfer function (MTF) this volume should be fully depleted and even over-depleted [6] in order to increase the carrier drift velocity and to minimize the charge diffusion during the drift time. High QE devices are normally back-side illuminated and made on very high resistivity (≈10 kΩ·cm) bulk silicon, however their normal operating voltages are not sufficient to achieve full depletion beyond approximately 50 µm. For example, the channel potential in Charge Coupled Devices (CCDs) is in the range 10–15 V, and in CMOS devices the diode bias rarely exceeds 5 V. To overcome this limitation, a separate reverse bias is applied across the substrate, and its magnitude depends on the resistivity and the thickness of the active semiconductor.

Reverse biasing is easily achievable with hybrid CIS or CCDs, but is a challenge for monolithic PPD devices due to the in-pixel well containing the readout transistors. One PPD example [7] achieves full depletion at the expense of adding another *pn* junction and significantly modifying the pixel. PPD layouts and doping profiles are usually highly optimized for good performance, and any changes should be carefully considered and simulated. Ideally, the structure of the PPD should not be modified when adding new elements for reverse biasing, and this has been our goal as well.

**Figure 1:**
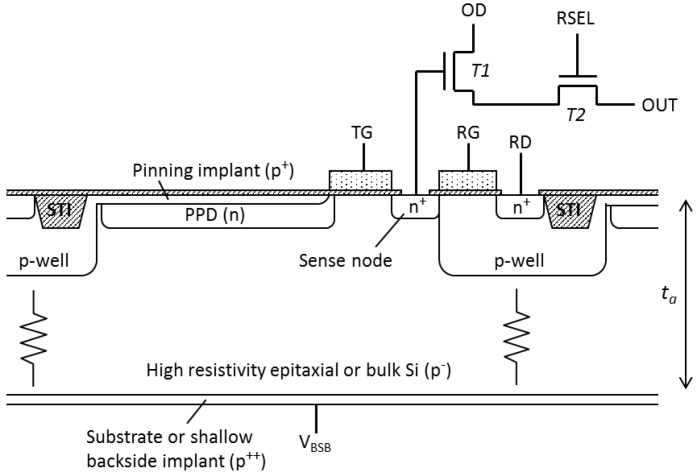
Cross section of a typical pinned photodiode (PPD) pixel showing the conductive path from the front-side p-wells to the p^++^ substrate (for front-side illuminated devices), or to the shallow p^++^ back side implant (for back-side illuminated devices). The transistors T1 and T2 are physically located in the p-well.

As shown in Figure 1 for n-type PPD, the front side p-wells are electrically connected to the p-type active layer. Since the p-wells are at device ground, applying negative substrate bias *V_BSB_* in order to increase the depletion depth under the PPD would result in large currents flowing from the front side p-wells to the backside p^++^ contact, as the p^+^/p^−^/p^++^ structure would conduct resistively.

If the leakage current can be eliminated, the full active thickness of the device $t_{a}$ can be depleted with the benefits of short charge collection time and good MTF. This paper describes a method for achieving this in a PPD CIS, details of the design and the results from the characterization of prototype devices, significantly expanding from the presentation given in [8].

## 2. Operating Principles

The main idea is to make all the individual depletion regions from adjacent PPDs merge under the p-wells, thus cutting off the parasitic front-to-back substrate current. This condition is not met naturally in PPD CIS due to the size and the depth of the p-well and the low PPD pinning voltage V_pin_, which is usually in the range between one and two volts.

**Figure 2:**
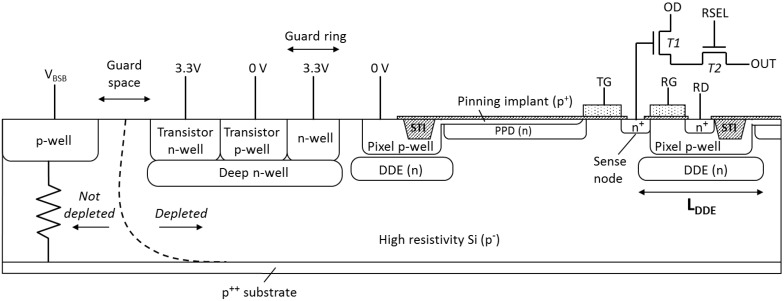
Cross section of the new design in a front-side illuminated (FSI) variant, showing the deep depletion extension (DDE) implant, the guard ring surrounding the pixel array, p-wells and n-wells for off-pixel transistors, and the edge p-well and its guard space for applying the reverse substrate bias from the front.

In the new design (Figure 2) an additional deep n-type implant is introduced under the pixel p-wells, while keeping the PPD structure unchanged. This implant has been dubbed “deep depletion extension” (DDE), does not connect to the PPD and has lower dopant concentration. In operation, the DDE becomes depleted and helps merge the diode depletion regions laterally under the p-wells, creating a pinch-off. The DDE regions acquire their potentials from the adjacent PPDs, and depending on their potential the DDE potential will vary across the device.

The size, shape, depth and doping concentration of the DDE were derived and optimized using commercial 2D and 3D TCAD tools. The goal was to create a potential distribution in which the DDE is less positive than the PPDs in order not to interfere with normal charge collection, while simultaneously forming a substantial potential barrier under the pixel p-wells to prevent parasitic flow of holes to the substrate. This is easier to achieve if the semiconductor resistivity and *V_pin_* are high because the size of the depleted region is approximately proportional to the square root of their product, using the considerations for an abrupt *pn* junction [9]. At the same time, the pixel p-well should be as shallow and narrow as possible in order to reduce the distance which has to be bridged.

Figure 2 shows some of the other important features in the design. A deep n-well is introduced to shield the transistor p-wells, and this allows both n-type and p-type Metal-Oxide-Semiconductor (MOS) transistors to be implemented on chip. The reverse substrate bias is provided from the front using the wide undepleted silicon along the chip periphery, and a guard space is provided to avoid junction breakdown. In backside illuminated (BSI) variant the substrate is ground away and a shallow p^++^ dopant is implanted into the back surface. The same metal pads are used for connections for both FSI and BSI variants, but for BSI chips the silicon around the pads is etched away for wire bond access from the back side.

**Figure 3:**
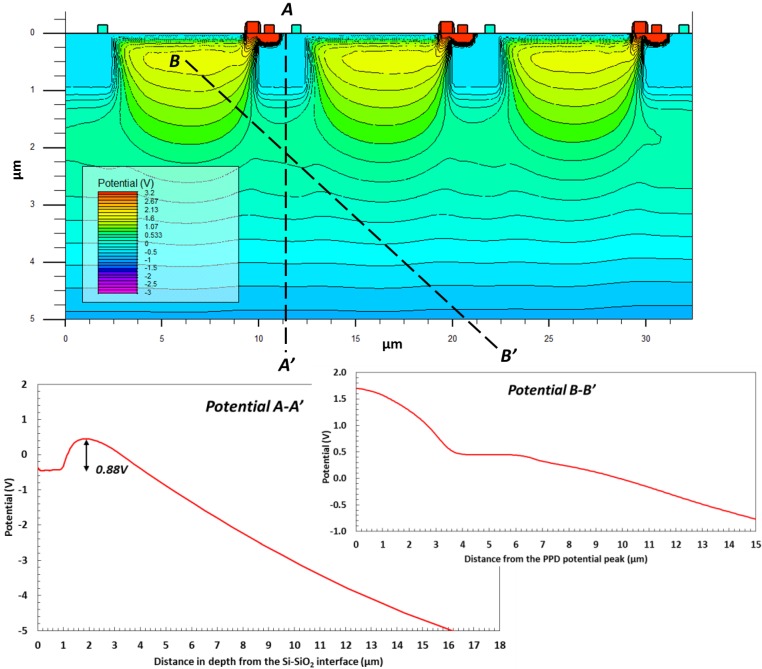
Potential distribution in a 2D simulation model reverse biased at −5 V, and the potential profiles along two lines: A–A’ is a vertical line crossing a p-well perpendicular to device’s surface; B–B’ starts from the potential peak in the PPD at 0.45 µm below the surface and cuts through the middle of the DDE implant. The two lines intersect at 2 µm along A–A’ and 5.25 µm along B–B’. The simulation is for 1000 Ω·cm, 18 µm thick epitaxial silicon and 10 µm pixels.

Figure 3 displays the potential distribution in a simplified 2D PPD model with DDE implants, containing 3 pixels. The line A–A’ shows the magnitude of the potential barrier under the pixel p-well and the potential down to the back side of the device. The line B–B’ illustrates the pinning voltage V_pin_ at the PPD center and also a region of low electric field (near zero potential gradient) in this particular direction. Simulations show that if the DDE doping is too high or its size is too large, the potential distribution under the p-well could form a shallow potential pocket. When the DDE region becomes more positive than the surrounding semiconductor some electrons get diverted to the DDE and eventually drained away without reaching the PPDs. On the other hand, too low DDE doping would not create the necessary potential barrier for prevention of substrate currents. Some parameter optimization is necessary to tune the behavior of the DDE, but this is greatly helped by the fact that the potential barrier can be adjusted electrically by the applied reverse bias.

**Figure 4:**
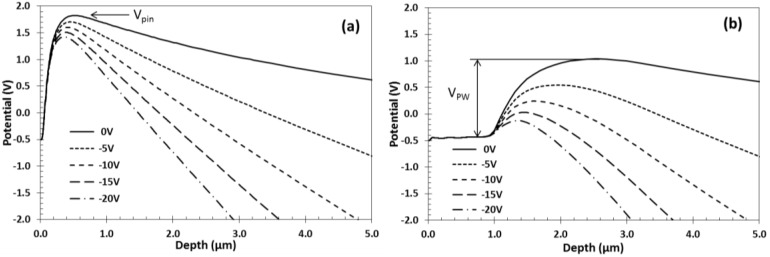
Simulated potentials through the center of a PPD (**a**); and though a p-well with DDE underneath it (**b**) as a function of the applied reverse bias *V_BSB_*. The simulation is for 1000 Ω·cm, 18 µm thick epitaxial silicon and 10 µm pixels.

Both the PPD potential and the barrier under the pixel p-well are influenced by the applied reverse bias because they are isolated space-charge regions and not tied to a fixed potential. Figure 4 shows that the barrier under the p-well *V_PW_* is influenced more strongly by the *V_BSB_* than the pinning voltage *V_pin_*. The reverse bias pulls down both potentials almost linearly, and from the data in Figure 4 we can calculate ${dV}_{pin}/{dV}_{BSB}=0.02$ and ${\mathrm{dV}}_{pw}/{dV}_{BSB}=0.056$. The stronger influence on *V_PW_* is due to the much greater depth of the DDE implant and its weak coupling to the PPDs, and is an important factor for device operation. The consequence of the decrease of *V_pin_* shown in Figure 4a is a reduction of the full well capacity (FWC), however this may not be detrimental if the output signal is limited by the sense node capacitance, as is typical for sensors with high conversion gain.

For 1000 Ω·cm, 18 µm thick epitaxial silicon and *V_pin_* = 1.8 V full depletion is achieved approximately at *V_BSB_* = −4 V, and further increase of the reverse voltage leads to over-depletion which can be beneficial for reducing the electron drift time and minimizing the Point Spread Function (PSF). Because most of the reverse bias drops across the active epitaxial silicon below the PPD, the principle of operation is applicable to much thicker sensor substrates, which require higher voltages for full depletion. Regardless of the thickness, the changes to the PPD and p-well potentials will be similar to the ones shown in Figure 4.

At sufficiently high reverse voltage the potential barrier below the p-well will be eventually reduced to a level where hole current will start to flow via thermionic emission [9]. However, if this occurs well above full depletion the condition will not have to be reached, and the device will function as desired.

The PPD potential is reduced when charge is collected, and since the barrier under the p-well is derived from the PPDs around it, the barrier will be reduced too. To make sure that this does not lead to undesirable signal-dependent substrate currents, the DDE has to be designed so that sufficient barrier height is maintained beyond full well capacity.

## 3. Prototype Device

**Figure 5:**
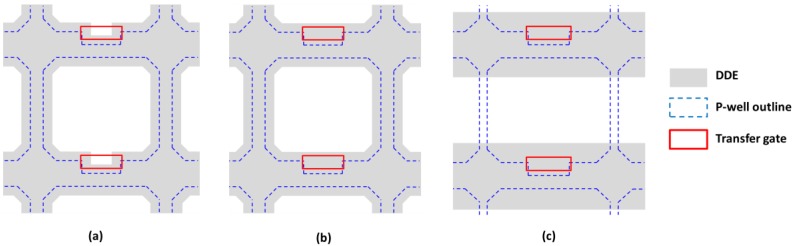
Simplified pixel layouts showing fully overlapping DDE over the p-well with (**a**) and without (**b**) notch around the sense node; and partially overlapping DDE (**c**). The sense node and the three transistors are not shown for simplicity.

A prototype device using the DDE concept was designed by us and manufactured by TowerJazz Semiconductor on a 180 nm image sensor CMOS process. The device, dubbed BSB1, uses 1000 Ω·cm 18 µm thick epitaxial silicon and contains eight arrays with 32 (V) × 20 (H) pixels each. Half the arrays are made of 10 µm square pixels and the other half of 5.4 µm pixels, with each array implementing different length of the DDE implant *L_DDE_*. In seven pixel variants the shape of the DDE follows the shape of the pixel p-well as shown in Figure 5a,b, and overlaps it in increasing steps. The shape without a notch around the sense node in Figure 5b was used when the design rules for high energy implants did not allow the manufacture of this narrow feature. In one of the 5.4 µm pixel variants the DDE only partially covers the p-well as in Figure 5c. This was deemed promising after significant narrowing of the p-well between the side edges of the PPD, which in simulation was able to achieve pinch-off naturally. Table 1 lists the design types used in the eight pixel arrays.

Three process variants with different depth of the DDE were manufactured: “shallow”, “medium” and “deep”, in combination with two different pinning voltages: 1.5 to 1.6 V (low *V_pin_*) and 1.7 to 1.8 V (high *V_pin_*). One wafer type with high *V_pin_* did not receive the DDE implant and was used as a reference, as the pixels on it did not receive any modifications.

Each row is selected by a simple combinational decoder controlling the row select gate and two analogue switches to the transfer and the reset gates. The outputs from the eight pixel arrays are multiplexed to common source followers and connected directly to chip pads. Only the middle 16 columns (out of 20) are read out.

**Figure 6:**
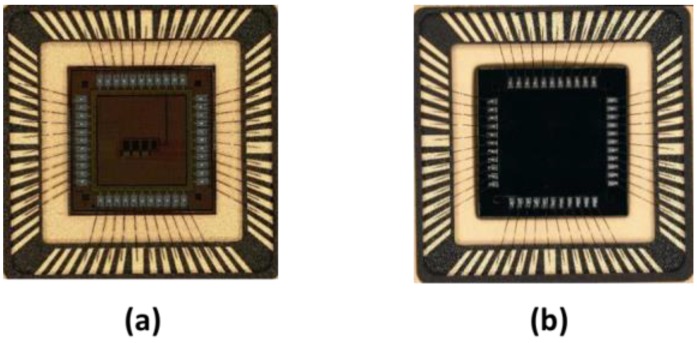
Photographs of the BSB1 chip: (**a**) front-side illuminated (FSI) variant; (**b**) back-side illuminated (BSI) variant. Die size is 5 mm × 5 mm.

BSB1 was designed to be compatible with backside thinning and illumination by using large bond pads and the biasing structure shown in Figure 2. Two wafers were processed by Teledyne e2v and thinned to 12 µm epitaxial thickness, with the silicon around the pads etched away for wire bonding from the back side, as is typical for such technology. The backside of the sensor was implanted with a shallow p^++^ dopant which passivates the surface and provides a low resistance path for the reverse bias to propagate across all of the device area. Figure 6 shows photographs of both BSB1 variants.

## 4. Experimental Results

### 4.1. Leakage Current under Reverse Bias in Darkness

The most important part of the sensor characterization was to establish that the DDE cuts off the leakage current under reverse substrate bias as intended. Initial results reported in [10] demonstrated that in all chip variants the reverse current was suppressed. The current was measured for the whole device and included the contributions from the eight pixel variants in parallel, together with the off-pixel digital and analog circuitry.

**Figure 7:**
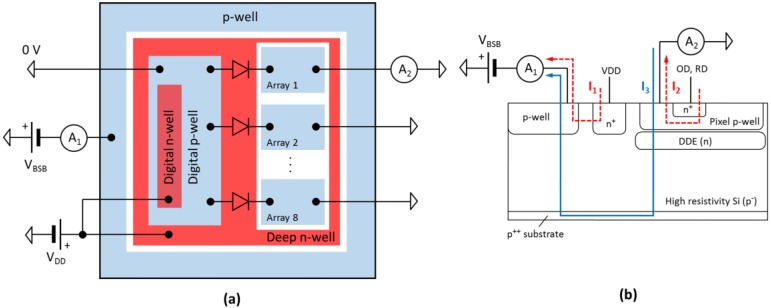
Simplified electrical diagram for the reverse current measurements (**a**) and the current components (**b**). The total substrate current *I_BSB_* is measured by the current meter A_1_ and the substrate current of the individual arrays by A_2_.

To characterize each DDE variant and measure its reverse current, the individual pixel arrays were designed with their own substrate connections. The pixel array substrates connect through diodes, part of the electrostatic discharge (ESD) protection, to the digital substrate as shown in Figure 7a. The effect is that the worst-performing pixel type can dominate the leakage current when its protection diode becomes forward biased, and this can limit the voltage applied to the whole chip.

**Figure 8:**
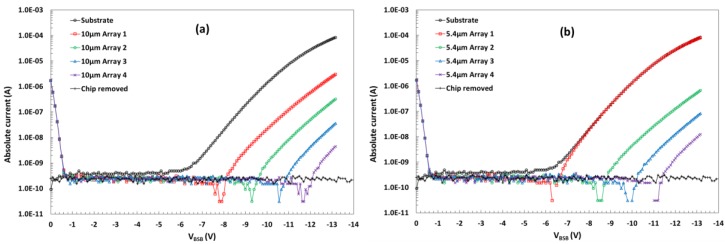
Substrate current for the whole chip and individual array substrate currents for 10 µm pixel (**a**) and 5.4 µm pixel variants (**b**) from a high *V_pin_*, medium DDE wafer. The absolute current values are plotted due to the change of sign of the pixel array currents. The plot with the chip removed indicates the sensitivity of the current measurement using the source measure unit model U2722A.

Figure 8 shows the experimentally measured total substrate current I_BSB_ and the individual substrate currents from the eight pixel arrays per chip. The I–V curves were taken while the sensor was continuously read out at 10 fps in darkness, and the current limits were set to 100 µA. This and all other measurements in this work were carried out at 23 ± 1 °C. At low *V_BSB_* values the two currents are dominated by the reverse *pn* junction currents *I*_1_ and *I*_2_ as illustrated in Figure 7b. At the threshold substrate voltage *V_BSB_* = *V_thr_* the potential barrier under the p-well is overcome and the substrate current *I*_3_ starts flowing in the opposite direction to *I*_2_. This leads to the change of sign in the current measured by A_2_, manifesting as “dips” in the I–V curves when the absolute current value becomes zero.

The dominant current is from the 5.4 µm array 1, which is the only one using the design in Figure 5c. The currents from the other seven arrays, which all use the fully overlapping design in Figure 5a or Figure 5b, are orders or magnitude lower. The length L_DDE_ (shown in Figure 2) increases in equal steps of 0.2 µm following the number of the pixel array.

Beyond the threshold voltage *V_thr_* the leakage current increases exponentially with the reverse voltage and can be described by thermionic emission of holes over a potential barrier, with an additional current $I_{2}$ from the reverse-biased *pn* junction. The absolute value of the array substrate current $I_{r}$ can be expressed from [9] as:

(1) $$\left|I_{r}\right|=\left|I_{3}-I_{2}\right|=\left|AT^{2}S\frac{m_{h}^{*}}{m_{0}}\exp\left(-\frac{V_{PW}+\beta V_{BSB}}{kT/q}\right)-I_{2}\right|,$$

where A is the Richardson’s constant for free electrons, S is the total area of the pixel p-well, $m_{0}$ is the free electron mass, $m_{h}^{*}$ is the effective hole mass in p-type silicon, $V_{PW}$ is the potential barrier height under the p-well at zero reverse bias, and β<0 is a proportionality coefficient describing the approximately linear decrease of the barrier height with increasing *V_BSB_*. In (1) the absolute value of *V_BSB_* is used even if in practice it is negative with respect to ground. Good estimates for $V_{PW}$ and β are obtained from the simulations in Figure 4b by fitting a straight line to the peak potential under the p-well as a function of *V_BSB_*. The absolute value of the threshold voltage can be expressed from (1) at the point when the thermionic current equals $I_{2}$ and gives

(2) $$\left|V_{thr}\right|=-\frac{1}{\beta }\left\{\frac{kT}{q}\ln\left(\frac{AT^{2}S}{I_{2}}\frac{m_{h}^{*}}{m_{0}}\right)-V_{PW}\right\}.$$

**Figure 9:**
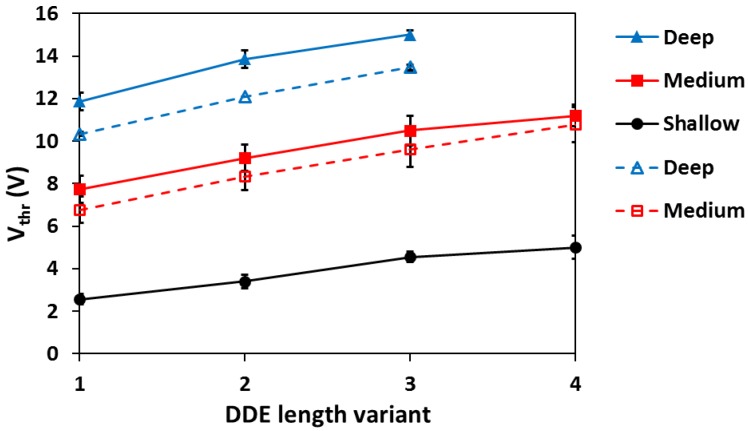
Experimentally measured threshold voltages in the 10 µm pixel variants with high *V_pin_* (closed symbols, solid lines) and low *V_pin_* (open symbols, dashed lines) for the three DDE depths. The error bars indicate the spread in *V_thr_* calculated from the available samples.

Figure 9 shows the measured threshold voltages in all of the 10 µm pixel variants. The experimental data indicates the trend for the *V_thr_* to increase with the length and the depth of the DDE implant, and qualitatively agrees with the TCAD simulations. However, a reliable quantitative comparison is difficult to obtain due to the several simplifications that had to be made in order to model a 3 × 3 pixel array in 3D.

In all pixel variants except those using the shallow DDE implant the threshold voltage exceeds 4 V even for the shortest L_DDE_. This result indicates that devices with medium and deep DDE can be over-depleted for both low and high *V_pin_*.

### 4.2. Electro-Optical Performance

#### 4.2.1. Photo Response

Electro-optical characterization was carried out by taking multiple images at increasing illumination levels, from which the photon transfer curves (PTC) [11] at different conditions are obtained. The PTC is a powerful characterization tool which can reveal hidden aspects of pixel operation through its sensitivity to deviations from the ideal Poisson noise distribution.

**Figure 10:**
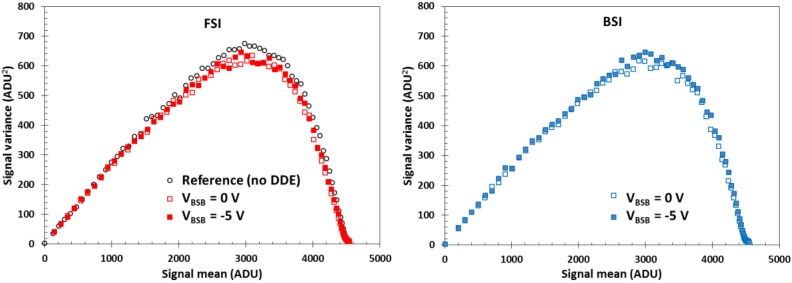
Photon transfer curves (PTCs) (“mean-variance” type with frame differencing) of a 10 µm pixel variant #1 in FSI and BSI implementation with high *V_pin_* and medium DDE. One ADU is 305 µV.

Figure 10 shows the PTCs obtained from the same 10 µm pixel design in FSI and BSI variants, together with the reference pixel which was made only in FSI variant. The five sets of data are virtually indistinguishable, and show identical charge-to-voltage conversion gain of 78 µV/e^−^ (obtained from the slopes at low signal values) and FWC. Applying reverse substrate bias beyond full depletion does not result in any detrimental effects on the characteristics of the sensor. In addition, measurements of the sensor’s linearity and image lag have shown that there is negligible difference between the reference and the new pixel designs, and that this does not change under reverse bias. The readout noise was below 5 e^−^ RMS.

Interestingly enough, even large substrate leakage currents reaching hundreds of microamps do not visibly change the photo response of the sensor. While the PPD is collecting electrons, the substrate current consists of holes, and the two carriers do not interact. Of course, in any practical application the substrate current must be kept to negligible levels or the reverse voltage applied to the sensor will be severely limited.

The results from the 5.4 µm pixel variants exhibit different characteristics and are discussed further on.

#### 4.2.2. Reverse Currents under Strong Illumination

When the PPD collects charge, the peak diode voltage decreases due to the compensation of the positively charged donor atoms by the stored electrons. The potential barrier created by the DDE is closely linked to the diode voltage, and it will decrease too. Under strong illumination this could cause the barrier to collapse and lead to a sharp increase of the reverse current. To investigate this, the reverse current of the whole chip and the individual pixel arrays were measured under high levels of illumination. The light stimulus was generated by a LED pulse immediately following a readout, thus ensuring that the collected charge is stored in the PPDs for the entire duration of the integration time. Because the integration time was much longer than the readout time (100 ms and 2 ms, respectively), this is equivalent to constant illumination with the desired intensity.

**Figure 11:**
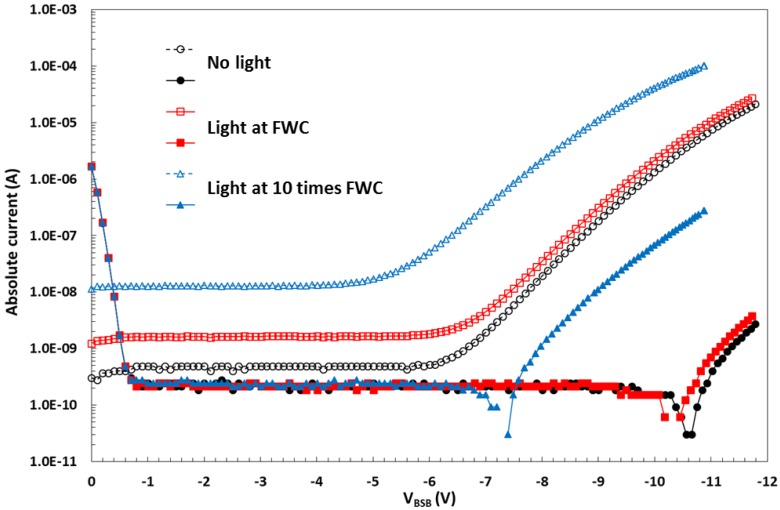
Whole chip (open symbols) and array substrate (closed symbols) currents in a 10 µm pixel design variant #3 with high *V_pin_* and medium DDE.

Figure 11 shows the measured currents in a 10 µm pixel variant and reveals a shift in the threshold voltage with increasing illumination levels. This is a clear indication that the potential barrier under the p-well decreases when charge is stored in the PPD, as expected. However, even at signal levels reaching 10 times FWC, *V_thr_* remains well above the voltage needed to maintain full depletion.

The chip area outside the pixel arrays is covered with deep n-well as shown in Figure 2 and is effectively a large reverse-biased *pn* junction. At low substrate voltages (below *V_thr_* of the worst-performing pixel variant) the substrate current of the whole chip should behave as in a typical photodiode, and increase linearly with the light flux. This is indeed observed in curves (b) and (c) in Figure 11, and can be used to verify the illumination levels beyond PPD saturation.

#### 4.2.3. Full Well Limiting 

**Figure 12:**
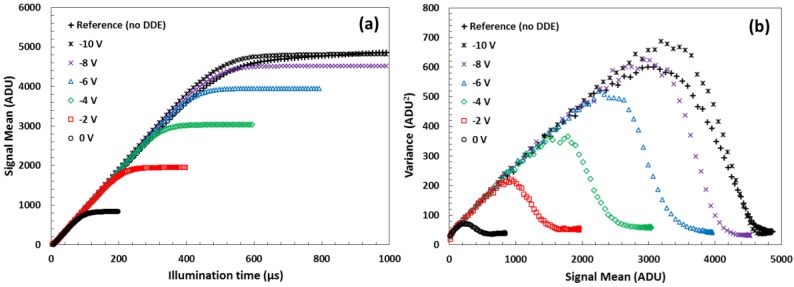
Photo response (**a**) and a PTC (**b**) of a 5.4 µm pixel variant #2 with low *V_pin_* and deep DDE for six *V_BSB_* values. The threshold voltage *V_thr_* for this pixel is 11.8 V.

In some 10 µm and in nearly all 5.4 µm pixel variants we observed significant reduction of the FWC under zero or low reverse bias, which fully or partially recovers once *V_BSB_* is increased sufficiently. The effect is mirrored in the PTC as well, and an example from a 5.4 µm pixel variant is shown in Figure 12. The FWC gradually increases until it is fully restored to the level seen in the reference pixels, and this occurs at a reverse bias below the threshold voltage. This “recovery” is observed in all 10 µm and most 5.4 µm pixel variants showing FWC limiting. The effect is more prominent in pixel variants with deeper DDE and lower *V_pin_*, as summarized in Table 2.

Simulations indicate that FWC limiting is caused by parasitic charge sharing between pixels, when the electrostatic potential in the DDE region is more positive than in the surrounding silicon at the same depth, and a potential pocket is formed as discussed in Section 2. Due to the mesh-like structure of the DDE implant, the collected charge can propagate to adjacent pixels and eventually gets drained away by the n^+^ guard ring terminating the pixel array next to the last DDE implant (Figure 2). The conditions for charge sharing can occur when the DDE doping concentration is too high and it cannot be fully depleted, or when its size is too large so that it begins to significantly overlap the PPD.

As shown in the simulation in Figure 4b, with increasing substrate bias the potential barrier under the p-well is lowered faster than the PPD pinning voltage and the DDE region becomes less attractive to electrons. At the same time, any potential pockets are gradually reduced and the mechanism for parasitic charge sharing is eliminated.

Under flat field illumination and FWC limiting the photo response exhibits a distinct roll-off at the edges of the image array, which is attributed to the hypothesized charge drainage by the n^+^ guard ring. The observed reduction of the photo response at the edges is approximately 20% and vanishes once the FWC is restored.

Due to design rule limitations for high energy implants, even the minimum length DDE occupies substantial part of a 5.4 µm pixel and extends over the p-well beyond the optimal implant size derived by simulations. This is largely avoided in the 10 µm pixels simply due to the difference in their size. As a consequence, most of the 5.4 µm pixel variants need reverse bias in order to eliminate the parasitic charge sharing.

### 4.3. X-Ray Response

Low energy X-rays generate well defined signal charges within the semiconductor volume, and offer an excellent characterization method for image sensors. This is used for measurements of the conversion gain, image lag, charge sharing and many other parameters.

**Figure 13:**
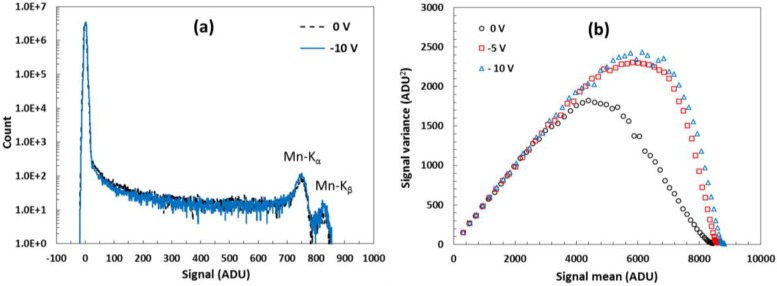
Data from a high *V_pin_*, deep DDE 10 µm pixel size variant #4: (**a**) ^55^Fe X-ray spectrum taken with *V_BSB_* = 0 V and −10 V; (**b**) PTC of the same pixel array showing FWC limiting. One ADU is 160.2 µV.

Figure 13 shows the acquired spectrum from a ^55^Fe source, emitting characteristic Mn-K_α_ (5.9 keV) and Mn-K_β_ (6.5 keV) X-rays, with a 10 µm pixel variant. The pixel exhibits approximately four percent lower FWC at *V_BSB_* = 0 V compared to −10 V, and its *V_thr_* is above −15 V. Despite the limited FWC showing in the suppressed signal variance, the X-ray spectra at the different substrate voltages differ very little, with a hint of higher fraction of split X-ray events at lower *V_BSB_*. The system gain calibration obtained from the X-ray spectrum was 2.1 e^−^/ADU, which matches well the gain calculated from the PTC (2.0 e^−^/ADU).

### 4.4. Depletion Depth Measurements and PSF

Provided that there is negligible leakage current, the chip design in Figure 2 ensures that the reverse bias reaches every part of the device. Nevertheless, additional measurements were carried out to verify this, and to establish that the device can indeed be fully depleted.

Despite the high resistivity epitaxial layer, the resistance of the front-to-back connection is low due to the 600 µm-wide edge p-well on a die with size 5 mm × 5 mm. The calculated resistance of 17 Ω is in a reasonable agreement with the measurement, which gave 21 Ω.

**Figure 14:**
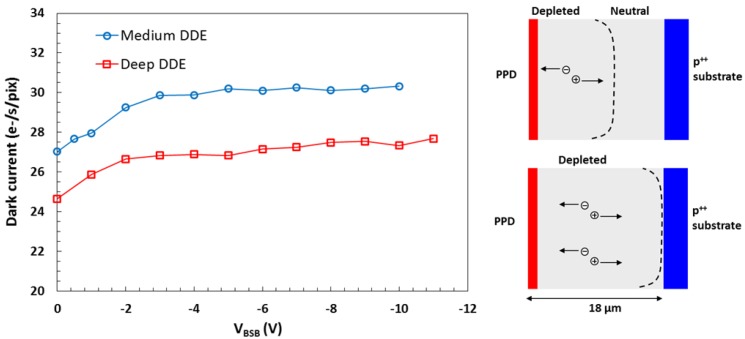
Dark current measurements in two FSI 10 µm pixels and a diagram illustrating the principle of the measurement.

In FSI chips the dark current is dominated by defects in the bulk silicon because the surface states are almost fully suppressed by the pinning implant [3], and the dark current increase is proportional to the depletion depth as shown in the diagram in Figure 14. Increasing the reverse bias beyond full depletion does not lead to further increase of the dark current, and the measurements in Figure 14 [10] from two 10 µm pixel variants confirm this. The results are in a good agreement with the expectation of achieving full depletion at *V_BSB_* = −4 V, based on the resistivity and the thickness of the epitaxial silicon.

**Figure 15:**
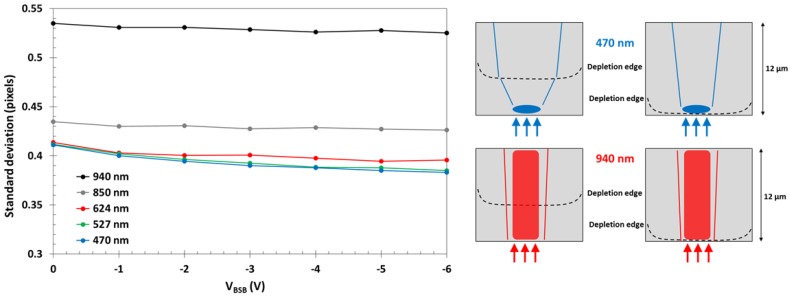
Measurement of the size of a spot illumination in a BSI device with high *V_pin_*, medium DDE at five wavelengths, and a diagram illustrating the illustrating the principle of the measurement. The diameter of the pinhole is 10 µm.

In BSI devices, the dark current is a factor of five larger, and is most likely due to imperfect passivation of the back surface. Because the dark current is no longer dominated by bulk silicon defects, the method used for the FSI devices is not feasible. Instead, spot illumination at different wavelengths using a precision pinhole placed on the back surface of the device was used. The size of the imaged spot is affected by charge diffusion and can be used as an indicator of the extent of the field-free region in the device, as shown in Figure 15 [8].

Short wavelengths (for example 470 nm, 0.6 µm absorption length in silicon) are absorbed near the back side, and if this region is field-free the charge will undergo some diffusion before collection. However, if the depletion reaches the back of the device there will be less diffusion, and the projected spot will appear sharper. Much longer wavelengths (for example 940 nm, 54 µm absorption length) are absorbed throughout the thickness of the device and the charge spread is much less sensitive to the extent of depletion, in particular in our relatively thin 12 µm BSI devices. From these considerations we expect the spot size to be less sensitive to the depletion depth (and by proxy on the reverse bias) as the illumination wavelength increases from 470 nm to 940 nm.

Figure 15 shows the measured spot sizes at five different wavelengths as a function of the reverse bias in a 10 µm pixel array. The cross section of the spot image in the horizontal direction was fitted with a Gaussian curve and the standard deviation is plotted.

The data is consistent with an increase of the depletion depth with the applied reverse bias V_BSB_. The size of the light spot levels off as the depletion edge reaches the back surface. The change of the spot size is less prominent for longer wavelengths, as discussed above.

## 5. Conclusions and Outlook

The characterization results from the newly developed reverse-biased PPD CMOS image sensor show successful operation. The device uses a new method for leakage current suppression by creating a “soft”, electrically adjustable potential barrier below the p-wells in the pixel by the introduction of an additional deep n-type implant. This allows reverse bias to be applied to the p-type substrate without undesirable leakage currents, so that the active semiconductor material can be fully depleted.

The operating principle creates new phenomena in PPD CIS such as signal-dependent leakage current which increases exponentially above a threshold reverse bias, and charge sharing effects. However, we demonstrate that when the device is correctly designed and operated these effects do not pose serious limitations. The new pixel design exhibits nearly identical performance to the traditional PPD pixel it is based on, with the very important difference that the active semiconductor material can be fully depleted and over-depleted.

This paper demonstrates an image sensor in an 18 µm thick epitaxial high resistivity silicon, but the principle of operation allows much greater depleted thicknesses to be achieved. The upper thickness limit is most likely to be determined by the practicalities of applying high voltage to the device and the deterioration of the MTF due to the increased carrier transit time. Full depletion of the sensor has been demonstrated in both FSI and BSI chips using two different methods.

This development has the potential to greatly increase the quantum efficiency of PPD CIS at near-IR and soft X-ray wavelengths, due to the potential to realize sensors with sensitive thickness in excess of 100 µm. Likely applications are in sensors for astronomy, machine vision, hyperspectral and high speed imaging, spectroscopy, microscopy and surveillance, as well as for consumer near-IR imaging. Soft X-ray (<10 keV) imaging at synchrotron light sources and free electron lasers is also likely to use this technology.

Future work will involve manufacturing of a large area CIS using one of the described pixel variants in a thicker epitaxial silicon, and also making a device on bulk silicon with thickness of at least 100 µm.

## Figures and Tables

**Table 1:** Summary of the pixel design variants with layouts shown in Figure 5.

Variant	10 µm	5.4 µm
1	a	c
2	a	b
3	a	b
4	a	b

**Table 2:** Pixel variants in which full well capacity (FWC) limiting has been observed are indicated by a gray-filled box.

	High *V_pin_*			Low *V_pin_*	
**10 µm**	**Shallow**	**Medium**	**Deep**	**Medium**	**Deep**
1					
2					
3					
4					
**5.4 µm**					
1					
2					
3					
4					

## References

[1] Fossum E.R., Hondongwa D.B. (2014). A Review of the Pinned Photodiode for CCD and CMOS Image Sensors. IEEE J. Electron Device Soc..

[2] Seo M.-W., Kawahito S., Kagawa K., Yasutomi K. (2015). A 0.27e^−^·rms Read Noise 220-µV/e^−^ Conversion Gain Reset-Gate-Less CMOS Image Sensor with 0.11-µm CIS Process. IEEE Electron Dev. Lett..

[3] Teranishi N. (2016). Effect and Limitation of Pinned Photodiode. IEEE Trans. Electron Devices.

[4] Teranishi N., Kohno A., Ishihara Y., Oda E., Arai K. (1984). An Interline CCD Image Sensor with Reduced Image Lag. IEEE Trans. Electron Device.

[5] Green M.A., Keevers M. (1995). Optical Properties of Intrinsic Silicon at 300 K. Prog. Photovolt..

[6] Holland S.E., Groom D.E., Palaio N.P., Stover R.J., Wei M. (2003). Fully Depleted, Back-Illuminated Charge-Coupled Devices Fabricated on High-Resistivity Silicon. IEEE Trans. Electron Devices.

[7] Resetar T., De Munck K., Haspeslagh L., Rosmeulen M., Süss A., Puers R., Van Hoof C. (2016). Development of Gated Pinned Avalanche Photodiode Pixels for High-Speed Low-Light Imaging. Sensors.

[8] Stefanov K.D., Clarke A.S., Ivory J., Holland A.D. Fully Depleted, Monolithic Pinned Photodiode CMOS Image Sensor *Using* Reverse Substrate Bias. Proceedings of the 2017 International Image Sensor Workshop.

[9] Sze S.M. (2006). Physics of Semiconductor Devices.

[10] Stefanov K.D., Clarke A.S., Holland A.D. (2017). Fully Depleted Pinned Photodiode CMOS Image Sensor With Reverse Substrate Bias. IEEE Electron Device Lett..

[11] Janesick J.R. (2007). Photon Transfer DN→λ.

